# Pulmonary Thromboembolism as a Potential Cause of Clinical Deterioration in COVID-19 Patients; a Commentary

**Published:** 2020-04-19

**Authors:** Saeed Safari, Mehdi Mehrani, Mahmoud Yousefifard

**Affiliations:** 1 Proteomic Research Center, Shahid Beheshti University of Medical Sciences, Tehran, Iran; 2 Emergency department, Shohadaye Tajrish Hospital, Shahid Beheshti University of Medical Sciences, Tehran, Iran; 3 Tehran Heart Center, Tehran University of Medical Science, Tehran, Iran; 4 Physiology Research Center, Iran University of Medical Sciences, Tehran, Iran

**Keywords:** COVID-19, Embolism and Thrombosis, Clinical Deterioration, Computed Tomography Angiography, Platelet Aggregation Inhibitors

## Abstract

Although the findings of some studies have been indicative of the direct relationship between the severity of clinical findings and imaging, reports have been published regarding inconsistency of clinical findings with imaging and laboratory evidence. Physicians treating these patients frequently report cases in which patients, sometimes in the recovery phase and despite improvements in imaging indices, suddenly deteriorate and in some instances suddenly expire. This letter aimed to draw attention to the role of pulmonary thromboembolism as a potential and possible cause of clinical deterioration in covid-19 patients.

**Dear editor:**

More than three months has passed from identification of the first case of pneumonia due to SARS-CoV-2 virus in China and it has subsequently spread to countries around the world. With publication of clinical, imaging, and laboratory findings of COVID-19 patients, our information on the behavior of the virus in the human body has increased.

Although the findings of some studies have been indicative of the direct relationship between the severity of clinical findings and imaging (1), reports have been published regarding inconsistency of clinical findings with imaging and laboratory evidence (2, 3). For example, a study on more than 1000 patients with COVID-19 in China has shown that about 18% of non-severe cases and 3% of severe cases had no abnormal finding in radiography and computed tomography (CT) scan (4).

In addition, significant and notable reports among the laboratory findings include thrombocytopenia, disturbances in coagulation profile [Prothrombin Time (PT), Partial Thromboplastin Time (PTT)], increase in D-dimer, and fibrin/fibrinogen degradation products (FDP). In a study on 94 patients, whose COVID-19 was confirmed using RT-PCR, Huan Han et al. showed that the serum levels of D-dimer, FDP, and fibrinogen (FIB) in these patients were higher than healthy individuals. They showed that D-dimer and FDP levels significantly and directly correlated with the severity of the disease and suggested D-dimer and FDP monitoring for early detection of severe cases (5).

Another study in China, which had evaluated about 300 patients, reported increased D-dimer levels in more than 35% of the studied patients. Based on the findings of this study, D-dimer levels were significantly higher in patients with more severe presentation of the disease (0.96 versus 0.35 mg/L; p < 0.001) (6). Findings of a study in Suzhou, China, has shown that fibrinogen level has increased in more than 65% of those with pneumonia due to COVID-19 and the interesting part is that at the level of 4.8 gr/L, the sensitivity of fibrinogen in differentiation of severe patients has been reported to be 100% (7). By studying 192 patients with COVID-19, Zhou et al. introduced D-dimer levels over 1 μg/mL as a predictor of poor outcome, which can aid physicians in detecting more severe patients in the initial stages (8).

In addition to the mentioned findings, physicians treating these patients frequently report cases in which patients, sometimes in the recovery phase and despite improvements in imaging indices, suddenly deteriorate and in some instances suddenly expire (9). 

Putting all the pieces of this puzzle together (thrombocytopenia, high serum D-dimer, inconsistency between clinical and imaging findings, and clinical deterioration of patients) a possible explanation for the afore-mentioned cases, from a clinical point of view, might be the formation of thrombosis. In this regard, a study revealed that CT angiography results of 25 patients with D-dimer levels higher than 6 μg/mL in China indicated pulmonary embolism in ten (40%) patients (10). In this study, the emboli were found to be centralized in the small branches of pulmonary artery. Additionally, using CT angiography, Yuanliang Xie et al. confirmed and reported the presence of pulmonary embolism in two COVID-19 patients who had deteriorated (11). 

Histopathologic findings of pulmonary biopsy of critical patients with COVID-19 pneumonia indicated small vessels hyperplasia, vessel wall thickening, lumen stenosis, occlusion, and micro-thrombosis formation. The blood vessels of alveolar septum were congested, edematous and widened, with modest infiltration of monocytes and lymphocytes, which could be additional evidence for confirmation of this hypothesis (12, 13).

A study on confirmed COVID-19 cases admitted to ICU reported a 31% prevalence of vascular thrombosis, which was most frequently (81%) seen in pulmonary vessels (14). 

It should be noted that micro-emboli are generally undetectable in CT angiography. Therefore, worsening of respiratory condition in hospitalized patients should raise high suspicion to thrombosis formation as a potential cause of clinical deterioration.

As a preliminary suggestion, it might be better to strongly consider performing pulmonary CT angiography for patients with disturbances in coagulation parameters, high D-dimer, pleuritic chest pain (reported in about 2% of patients) (15), inconsistency of dyspnea with imaging findings, as well as those whose clinical symptoms worsen and deteriorate during treatment. We should also add anticoagulant agents to the treatment cocktail of high-risk patients such as old, obese, pregnant, and intubated cases, as well as those with other risk factors of thromboembolism.
